# Synthesis of Cu and CuO nanoparticles from e-waste and evaluation of their antibacterial and photocatalytic properties

**DOI:** 10.1007/s11356-023-28437-5

**Published:** 2023-07-17

**Authors:** Sabah M. Abdelbasir, Diaa A. Rayan, Mahmoud M. Ismail

**Affiliations:** 1 Central Metallurgical R&D Institute (CMRDI), P.O. Box 87, Helwan, Cairo, 11421 Egypt; 2 Department of Physics, Deraya University, New Minya, Minya, Egypt; 3 Physics Department, Faculty of Science, Al-Azhar Unversity, Nasr City, Cairo, 11884 Egypt

**Keywords:** Copper nanoparticles, WPCBs, Ammoniacal leaching, Rhodamine B, Photocatalytic, Antibacterial activity

## Abstract

Waste printed circuit boards (WPCBs) contain a plethora of valuable metals, considered an attractive secondary resource. In the current research, a hydrometallurgical process combined ammonia/ammonium chloride leaching and reduction (using L-ascorbic acid) to recover copper and its oxide (CuO) as nanosized particles from WPCBs was investigated. The results of leaching indicated that 96.7% of copper could be recovered at a temperature of 35 °C for a leaching duration of 2 h with ammonium chloride and ammonia concentration of 2 mol/L at a solid:liquid ratio of 1:10 g/cm^3^. The synthesized particles exhibit spherical and distorted sphere morphology with average particle size of 460 nm and 50 nm for Cu and CuO NPs, respectively. The antibacterial activity of Cu, CuO, and a (1:1) blend of both (Cu/CuO) has been examined against five different bacterial and fungal strains. The highest zone of inhibition was measured as 21.2 mm for Cu NPs toward *Escherichia coli* and 16.7 mm for Cu/CuO blend toward *Bacillus cereus* bacteria. The highest zone of inhibition was measured as 13 mm and 13.8 mm for Cu/CuO blend toward *Fusarium proliferatum* and *Penicillium verrucosum* fungi. Cu/CuO blend showed notable photocatalytic activity towards Rhodamine B dye under visible light irradiation with 96% degradation rate within 120 min. Using the process developed in this study, copper and its oxide as nanoparticles can be produced from WPCBs and used for multifunctional applications.

**Graphical abstract:**

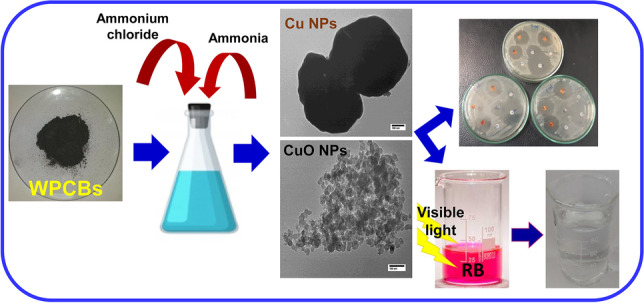

**Supplementary Information:**

The online version contains supplementary material available at 10.1007/s11356-023-28437-5.

## Introduction

A major challenge and opportunity of mining municipal waste is the management of waste electrical and electronic equipment (WEEE) (Abdelbasir et al., 2018a; Mdlovu et al., 2018). According to estimates, the total generation of these wastes is currently around 52.20 million tonnes (Abdelbasir et al., 2020) that will rise to reach 74.7 million tons in 2030 (Forti et al., 2020; Seif El-Nasr et al., 2020). WPCBs (waste printed circuit boards) account for approximately 10% of WEEE total production (Abdelbasir et al., 2018b). WPCBs contain approximately 30% of their total weight in metals such as copper, iron, tin, nickel, lead, zinc, silver, gold, and palladium, and as a result, its waste is regarded as a source of funds for urban metal mining (Abdo et al., 2021; Huynh et al., 2020; Tatariants et al., 2018).

Various recycling practices involving pyrometallurgy, hydrometallurgy, or combination of the two have been investigated in conventional approaches (Cucchiella et al., 2015; Prasad et al., 2020). High energy consumption, large gaseous emissions (as furans), and large waste or slag generation have been identified as problems accompanied with pyrometallurgical methods (Ádám et al., 2021; Pathak et al., 2017). Hydrometallurgical recycling, on the other hand, necessiated a large amount of chemicals. The unconsumed chemical discharge with effluent volume is a serious environmental threat (Borthakur and Singh, 2017). As a result, significant efforts have been made to develop the environmentally-friendly processing of e-waste.

Generally, nanomaterials have a specific field of application due to their unique properties, such as copper nanoparticles (Cu NPs) for high thermal conductivity, high-strength alloys, and antibacterial and antiviral compounds (Aguilar et al., 2019; Akhavan and Ghaderi, 2010; Das Jana et al., 2021; Le et al., 2023; Lei et al., 2013). Cu NPs are also widely used in a variety of fields, including photochemical catalysis, electronics, optics, biosensing, and gas sensors (Akhavan and Ghaderi, 2011; Khodashenas and Ghorbani, 2014; Patil et al., 2015; Tadjarodi et al., 2015). Cheap and abundant, cupric oxide (CuO) has a small band gap (*E*_g_ = 1.2 eV) and is widely used as a semiconductor in various fields such as catalysts, electrochemicals, energy storage, and chemical transformations (Sonia et al., 2015; Verma and Kumar, 2019; Xue et al., 2017; Yan et al., 2015). Recently, some attempts were made to produce Cu-containing nanoparticles from WPCBs, for example, using electrokinetic, thermal micronizing, supercritical methanol, and green bio-inspired synthesis processes (Abdelbasir et al., 2020; Dabhane et al., 2023; Shokri et al., 2017; Xiu et al., 2017). Green methods are considered eco-friendly and cost-effective techniques for producing nanoparticles to be used in various applications (Daphedar et al., 2022; Hassanisaadi et al., 2021; Rahdar et al., 2020)

Different types of microorganisms cause problems in living conditions and have serious implications for health care. Increasing antibiotic resistance has sparked a lot of studies to overcome challenges in various fields including small antibiotics, cationic polymers, metal nanoparticles, and antimicrobial peptides (Akhavan et al., 2011; Ananth et al., 2015; Song et al., 2011; Zhou et al., 2020). Metal nanoparticles have been extensively studied because they possess a variety of instinctive antimicrobial mechanisms, such as disruption of the cell membrane; diffusion into and degradation of internal cellular components such as DNA, RNA, and enzymes; and the release ions with antimicrobial activity (Dizaj et al., 2014). There are a variety of materials available, including silver, gold, copper, zinc, and their corresponding oxides (Ingle et al., 2014).

The US Environmental Protection Agency (EPA) has recognized copper and its compounds as antimicrobial materials (Arendsen et al., 2019). Copper and its oxides (i and ii) in the nanosize (less than 100 nm) display enhanced antimicrobial activity towards pathogenic microorganisms (Elsayed et al., 2020; Tatariants et al., 2018). Numerous studies have been conducted to investigate the antibacterial activities of elemental Cu and its oxides in relation to particle size (Chen et al., 2019), morphology (Chen et al., 2021), and dissolution of copper ions in different media (Alagarasan et al., 2021)*.*

Copper and its oxides are considered also as the most attractive photocatalysts for the photodegradation of organic pollutants due to their low fabrication cost, high optical absorption, and optimal optical band gap for visible driven photocatalytic activity (Katal et al., 2018; Lu et al., 2015). They are well capable to absorb visible light and generate electron-hole pairs thus involving a chemical reaction with the organic pollutant (Mosleh et al., 2018; Sorekine et al., 2022). In addition, they are readily available, have superior charge separation abilities, better chemical stability, are non-toxic, and are easily shaped in a variety of shapes and sizes (Wang et al., 2021). In the last years, remarkable progress have been made in the photodegradation of dye pollutants under ultraviolet and visible light (Sinha and Ahmaruzzaman, 2015; Sundararajan and Kennedy, 2017). Nevertheless, for better photocatalytic performance, a combination of both CuO and Cu_2_O with Cu improved their photocatalytic (Mosleh et al., 2018; Sahoo et al., 2016) degradation performance toward dyes. Jiang et al. (2017) synthesized the CuO–Cu_2_O powder and investigated the effect of CuO morphology on the photocatalytic properties.

Herein, a simple, low-cost method for recovering copper and its oxide (CuO) as nanosized particles from WPCBs is presented. Copper is first retrieved from WPCBs by ammoniacal–ammonium chloride solution. The impact of various parameters affecting the copper recovery such as leaching time, temperature, solid/liquid ratio, and concentrations of the leachant solution is investigated. L-ascorbic acid has been proposed as a reductant as well as a stabilizing agent. Structural characteristics of the prepared NPs were examined by X-ray diffraction (XRD), field emission scanning electron microscopy (FESEM) equipped with energy dispersive X-ray spectroscopy (EDX), and transmission electron microscopy (TEM). Antimicrobial activities of the prepared NPs were examined by a well disk diffusion assay and minimum inhibitory concentration (MIC) of the NPs against various bacterial strains. Photocatalytic activity of the NPs to break down Rhodamine B (Rhod-B) dye was investigated as well.

## Experimental work

### Materials

All of the chemicals used were of the highest purity. Ammonium chloride (NH_4_Cl; Alfa Aesar) was dissolved in 10% ammonia solution (NH_3_; 25% Adwic Co., Egypt) to prepare the leachant solution. For the preparation of Cu and CuO NPs, L-ascorbic acid (C_6_H_6_O_8_; Alpha Aesar) was used as a reductant and cetyltrimethylammonium bromide [(C_16_H_33_)N(CH_3_)_3_Br, CTAB]; Sigma-Aldrich] as a crystal modifier. Rhodamine B dye was used for testing the photocatalytic degradation of the prepared particles and pure water was used throughout all experiments.

A local computer shop provided about 2 kg of WPCBs from old computers that were sliced to pieces of about 5 cm^2^. Using a laboratory-scale crusher, the slashes of the WPCBs were crushed, and then ground to 0.5 mm in a disk mill (HERZOG Maschinenfabrik GMBH Co.). Chemical composition of WPCBs was determined via X-ray fluorescence spectrometer (XRF) (Axios Advanced WDXRFP analytical, Netherlands) and confirmed by atomic absorption spectrometer (Savantaa, Australia). Table 1 shows the main element content of the WPCB sample.

**Table 1:** Analysis of WPCBs determined by XRF

Element	Cu	Sn	Pb	Fe	Ni	Au	Ag	Zn	Cr	Mn	Ti	Others
Content (wt%)	22.00	9.50	1.56	2.40	0.61	0.07	0.13	0.46	0.07	0.04	0.60	62.56

### Leaching experimentations

WPCB leaching experiments with ammoniacal ammonium chloride were performed in a 500-mL double-necked glass reactor mounted with a condenser and a thermometer for temperature control. The reactor was dipped in a water bath on a stirring hotplate and stirring rate was kept constant at 400 rpm. Figure S1 in the supplementary file depicts a drawing of the used system. The reactor was loaded with leachant solution (concentration range of 0.5 to 2.0 M), followed by 10 g of WPCB powders. In all experiments, 20 mL of leaching solution was used with a *L*/*S* ratio of 10 except where specified. Temerature (25–80 °C) and alkaline salt concentration (0.5–2 M) variations were considered. Experiments with *L*/*S* of 20 and 30 were also carried out to determine the best conditions. Residue after leaching was filtered and thoroughly rinsed with water and metal concentrations in the filterae were measured using an atomic absorption spectrometer (AAS). The copper recovery percentage was calculated using mass balancing after analyzing the raw WPCB powder and the leach residue. For the optimal conditions, experimentations with *L*/*S* of 20 and 30 were also conducted. The leaching residue was filtered and rinsed carefully with pure water. Atomic absorption spectrometer (AAS) was used for analyzing metal concentration in solutions. Copper recovery is defined as the percentage of copper leached into solution from the raw sample, as calculated by the following equation:

1 $$Copper\ recovery\ \left(\%\right)=\frac{Copper\ leached\ in to\ solution}{Total\ copper\ in\ WPCB\ original\ sample}\times 100$$

### Synthesis of copper and coppper oxide nanoparticles

To prepare Cu NPs, a suitable weight of CTAB (0.01 g) was dissolved in 10 mL water, followed by the addition of 20 mL of leached copper solution and L-ascorbic acid, and the solution was heated at 70 °C for 30 min (Fig. S2 displays the used system for naanoparticle synthesis). The solution was then allowed to cool overnight before being filtered, washed repeatedly with pure water and ethanol, and dried under vacuum..

**Fig. 1:**
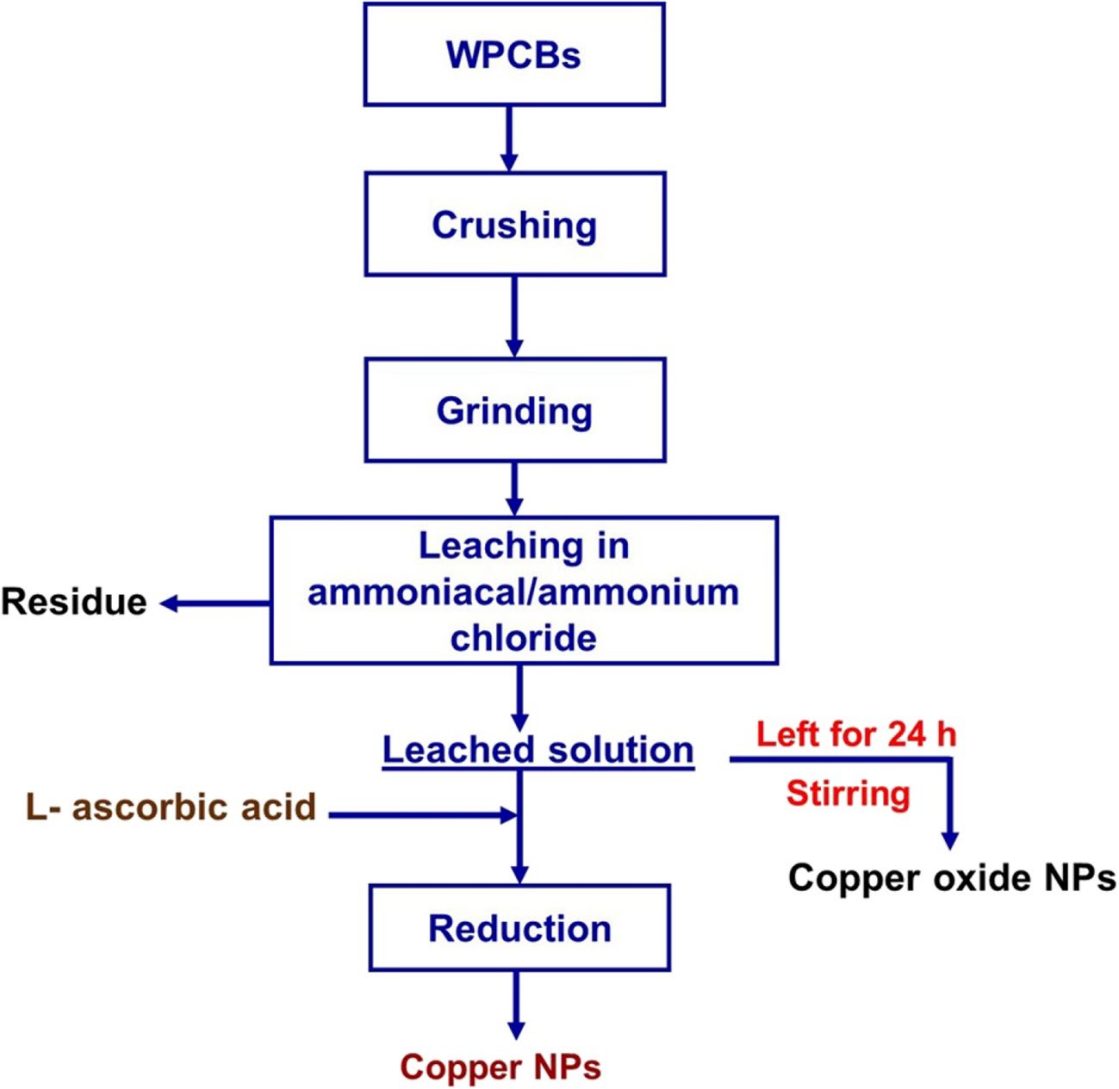
A schematic representing the route of recovering copper and copper oxide nanoparticles from WPCBs

For copper oxide nanoparticle (CuO NP) production, 10 g of WPCB powder was leached for 3 h with NH_4_Cl (0.5–2.0 M) in 10% ammonia solution (*S*/*L* ratio 1/10). The solution was stirred constantly for 2 h and then left overnight. The formed CuO powder was centrifuged, washed with pure water and ethanol, and dried. Figure 1 displays the full procedure of the NP synthesis. A sample of both particles (1:1 ratio) was blended, and its photocatalytic and antibacterial properties were evaluated and compared with the fabricated pure nanoparticles.

### Material characterization

Composition and features of the produced nanoparticles (Cu, CuO, and Cu/CuO blend) were confirmed by different characterization tools as fully described in the supplementary file.

### Photocatalytic activity evaluation

**Fig. 2:**
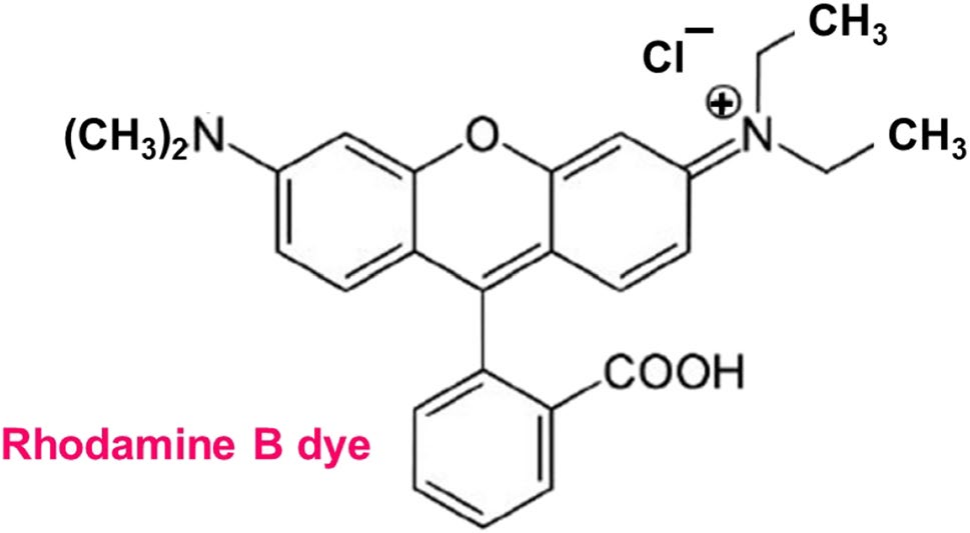
Molecular structure of Rhod-B dye

The photocatalytic action of Cu/CuO NPs was assessed by the degradation of Rhod-B dye in an aqueous solution at different periods (0–120 min). Stock solution (10 mg/L) of Rhod-B was prepared (Fig. 2). In the experiment, 10 mg of Cu/CuO NP blend (ratio 1:1) was mixed with 100 mL of Rhod-B solution (10 mg/L) and pH was adjusted to 9.0 in the dark at normal temperature (Mali et al., 2020). The suspension was then treated with ultrasonic waves for 10 min and magnetically stirred for 60 min in the dark to attain adsorption-desorption equilibrium. Following that, the solution was placed under visible light using Luzchem LZC 4V (Canada) multilamp photoreactor with regular stirring.

About 3-mL aliquot of the suspension was taken and centrifuged at selected time intervals to remove Cu/CuO NPs. A UV-Vis spectrophotometer was used to measure the absorption spectrum, which was used to calculate the rate of dye degradation. The efficiency of photocatalytic degradation was calculated using the next equation:

1 $$Degradation\ efficiency\ \left(\%\right)=\frac{C-{C}_0\ }{C_0}\times 100$$

where *C*_0_ stands for the initial dye concentration and *C* stands for the reminant dye concentration after time *t*.

### Antibacterial activity testing

The inhibitory effect of Cu and CuO NPs was carried out on five strains of pathogenic bacteria: two gram positive, namely, *Bacillus cereus* EMCC 1080 and *Staphylococcus aureus* ATCC 13565, and three gram negative, namely, *Salmonella typhi* ATCC 25566, *Escherichia coli* 0157 H7 ATCC 51659, and *Pseudomonas aeruginosa* NRRL B-272. Stock cultures were grown on nutrient agar slant for 24 h at 37 °C and then refrigerated until use. Also, five fungal species were used for antifungal assay: *Aspergillus flavus* NRR 3357, *A. ochraceus* ITAL 14, *A. niger* IM I288550, *Fusarium proliferatum* MPVP 328, and *Penicillium verrucosum* BFE 500. The stock cultures were grown on potato dextrose agar slant at 25 °C for 5 days before being stored in the refrigerator until use.

An inoculum of bacteria was inoculated into a 5-mL tube of tryptic soy broth after 24 h of incubation on nutrient agar slants of each bacterial species. The broth culture is incubated at 35 °C for 4 h until it reaches the McFarland BaSO_4_ turbidity standard of 0.5 (10^8^ cfu mL^−1^). The sensitivity tests of Cu and CuO NPs were performed on various bacterial cultures using disk diffusion method by Kirby-Bauer technique (Bauer et al., 1966; Marrez et al., 2019). The negative control was DMSO and ceftriaxone (1 mg mL^−1^) was the positive control. The inoculated plates were then incubated at 37 °C for 24 h. Inhibition zones were then measured and expressed as the diameter of the clear zone plus the diameter of the paper disk. The fungi were grown on potato dextrose agar (PDA) for 5 days at 25 °C. Each fungus’ spore suspension was prepared in 0.01% Tween 80 solution. When the fungal suspension was compared to the 0.5 McFarland standard, the turbidity of the inoculum suspension represented approximately 2 × 10^8^ cfu mL^−1^. A negative control was made with DMSO and a positive control was made with the commercial fungicide miconazol (1 mg mL^−1^). The inoculated plates were incubated for 24–48 h at 25 °C and the antifungal activity was assessed by measuring the zone of inhibition (mm) against the tested fungus (Medeiros et al., 2011). All treatments had three replicates, and the experimental results were averaged.

## Results and discussion

### Material preparation and characterization

To preferentially retrieve copper from the WPCBs, ammonium salt reagent would react specifically with copper ions forming stable complexes at alkaline pH (9.0–11.0). As a result, by leaching with ammoniacal ammonium chloride leachant, it can be retrieved from other metals in WPCBs. During the process, ammonium chloride provides anions to the copper ammine complex [Cu (NH_3_)_n_^2+^] as well as H^+^ to react with OH^−^ anion freed during the reaction as follows (Yoo and Kim, 2012):

2 $${NH}_4 OH\to {NH}_3+{H}_2O$$

3 $${NH}_3+{H}_2O\to {NH_4}^{-}+ OH$$

4 $$Cu+2{NH}_3\cdot {H}_2O+2{NH}_4 Cl+\frac{1}{2}{O}_2\to \left[ Cu{\left({NH}_3\right)}_4\right]{Cl}_2+3{H}_2O$$

Many variables influence copper leaching or retrieval such as ammonium chloride’s concentration, leaching temperature, and *S*/*L* ratio. Ammonia solutions are highly specific and they are a cost-effective choice for dissolving specific metals. Furthermore, its cost is low in comparison to many other solvents (Sun et al., 2015).

**Fig. 3:**
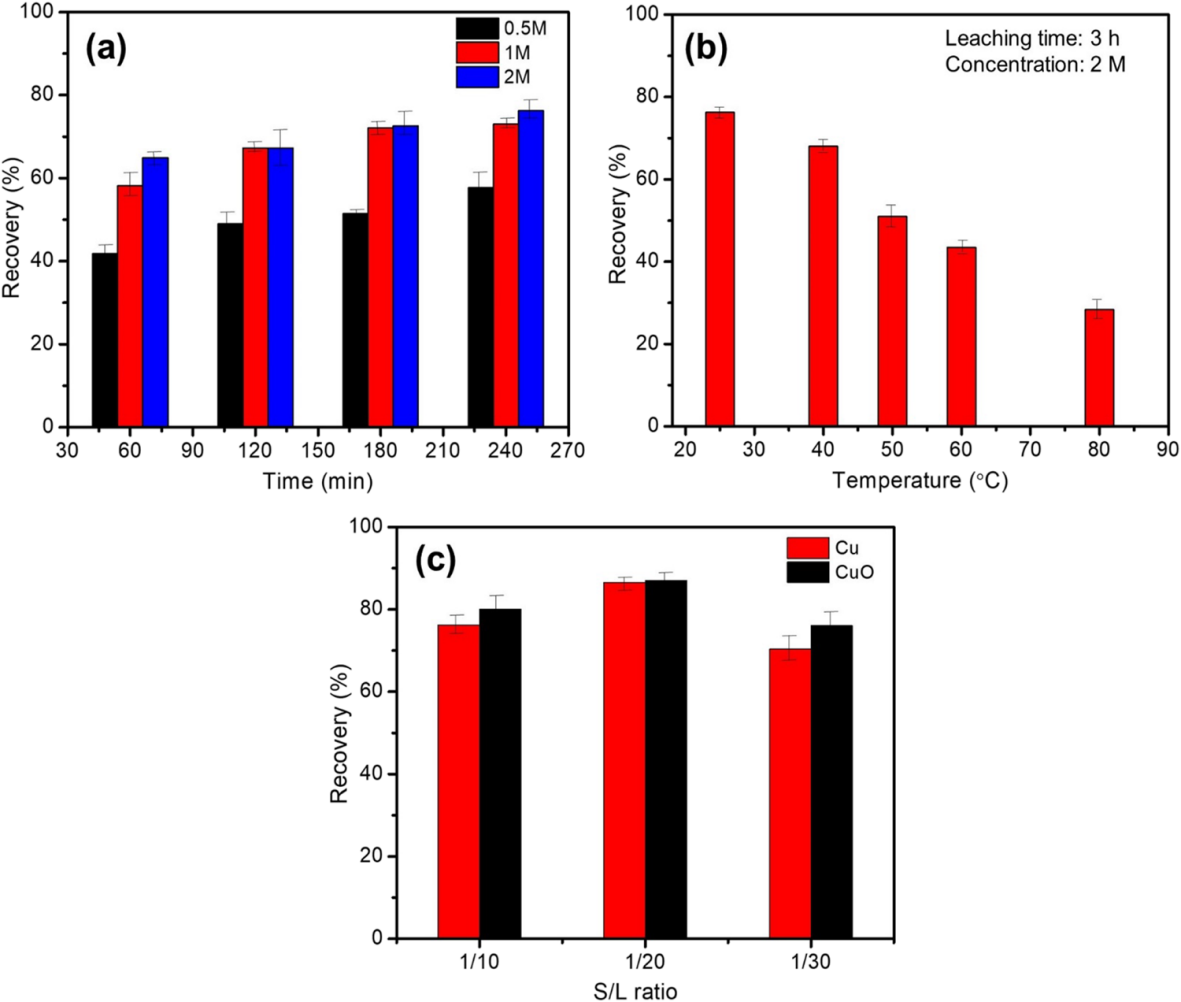
Effect of **a** leaching time, **b** temperature, and **c** *S*/*L* ratio on the copper recovery percentage using ammonium chloride (2M), ammonia concentration of 8%, and constant stirring rate of 400 rpm

Copper leaching increases as leachant concentration increases over the same leaching period (Fig. 3(a)). According to Eq. (4), the theoretical amount of ammonia solution for leaching is when the concentration reaches 2 mol/L. When the concentration of leachant is 0.5 mol/L, the highest copper recovery reaches 74% after 3-h leaching time. With the increase of the leachant concentration to 2 mol/L, the maximum recovery of copper can reach 78.90%. This enhanced dissolution could be linked to the increased amount of (NH_4_^+^) as ligand in the leaching medium to form the stable cuprammine complexes (Liu et al., 2010; Seif El-Nasr et al., 2020). Temperature can have a significant impact on ammonia vaporization in the leaching medium. Ammonia losses due to vaporization from solution would occur at high temperatures (50–80 °C), due to the high vapor pressure and volatility of the solution (Shi et al., 2022). The copper recovery percentage would be affected by these ammonia losses through the retrieval process decreasing from 78% at 25 °C to 30% at 80 °C.

The solid-to-liquid (*S*/*L*) ratio could also affect copper recovery from leachant solution. The leachant’s volume is increased while the concentrations of ammonia and ammonium salt are maintained constant at ambient temperature and constant stirring. As seen from Fig. 3(c), an increase in *S*/*L* ratio from 1:10 to 1:20 considerably increases the recovery of Cu and CuO from 78 to 85% and from 80 to 87%, respectively. As the *S*/*L* ratio approaches 1:30, retrieval decreases noticeably, possibly because the pulp consistency is reduced, aiding the diffusion of reactants and products in less liquid-to-solid ratios (Seif El-Nasr et al., 2020). Also, an increase in the *S*/*L* ratio raises the initial concentration of leachant and promotes higher mass transfer, which promotes the formation of cuprammine complexes (Pinho et al., 2021).

**Fig. 4:**
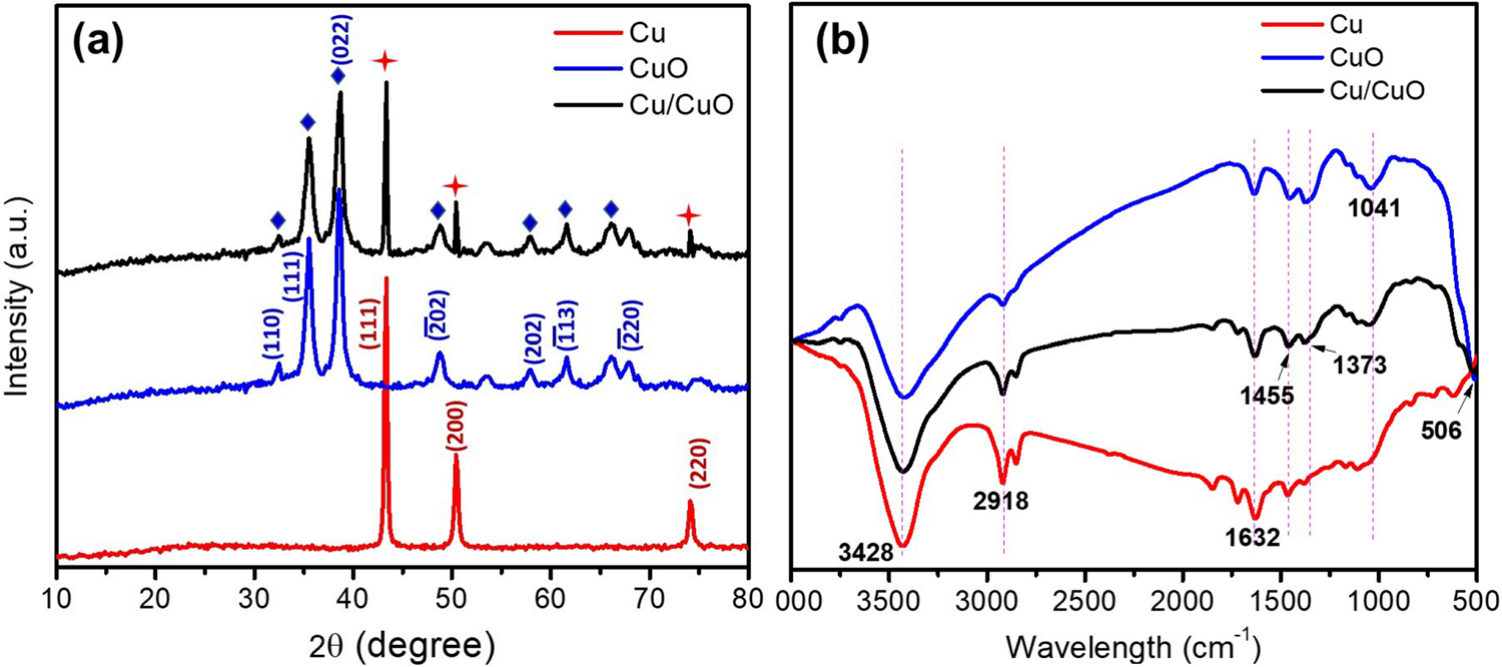
**a** XRD patterns and **b** FTIR spectra of Cu, CuO, and Cu/CuO nanoparticles

The structural composition of the produced Cu and CuO NPs was recognized from XRD pattern presented in Fig. 4(a). Bragg reflections at 2θ values 43.047°, 50.189°, and 73.910° characterize the crystallographic planes (111), (200), and (220) of cubic structure of copper (JCPDS card no. 85-1326). XRD patterns for CuO NPs validated its high purity according to the standard card (JCPDS 48-1548). According to the strong peaks in the XRD patterns, the CuO NPs were crystalline with no impurities. The broadening of the peaks indicates that the average crystal size is small (22 nm), as predicted by peak (111) and (022) using the Scherer formula (Nayak et al., 2019).

5 $$D=\frac{K\lambda}{\beta \cos \varTheta }$$

where *D* is the mean particle size, *k* (is a constant) = 0.9, *λ* is the wavelength of X-ray source (0.1541 nm), *β* is the full width at half maximum (FWHM), and *θ* is the half diffraction angle. According to the well-known Scherrer equation, the average crystal size for Cu NPs was calculated to be 41.7 nm.

FTIR spectroscopy was utilized to define the produced NPs, as represented in Fig. 4(b). A peak at 506 cm^−1^ correlated to the Cu–O bond vibrations was observed confirming the presence of CuO NPs (Karuppannan et al., 2021; Kuppusamy et al., 2017). Strong and broad band at 3428 cm^−1^ corresponds to the stretching (O–H) of adsorbed water (Ananth et al., 2015b). The peak at 1041 cm^−1^ specified the occurrence of C–O stretching (Fuku et al., 2020), whereas the peak at 1632 cm^−1^ signified the C = C stretch vibrations (Yousef et al., 2018). Furthermore, broad vibrational stretches were also recognized at 2918 cm^−1^ and 1455 cm^−1^, which were primarily credited to the adsorbed H_2_O molecules on the surface of the synthesized particles (Ananth et al., 2015; Yousef et al., 2018).

**Fig. 5:**
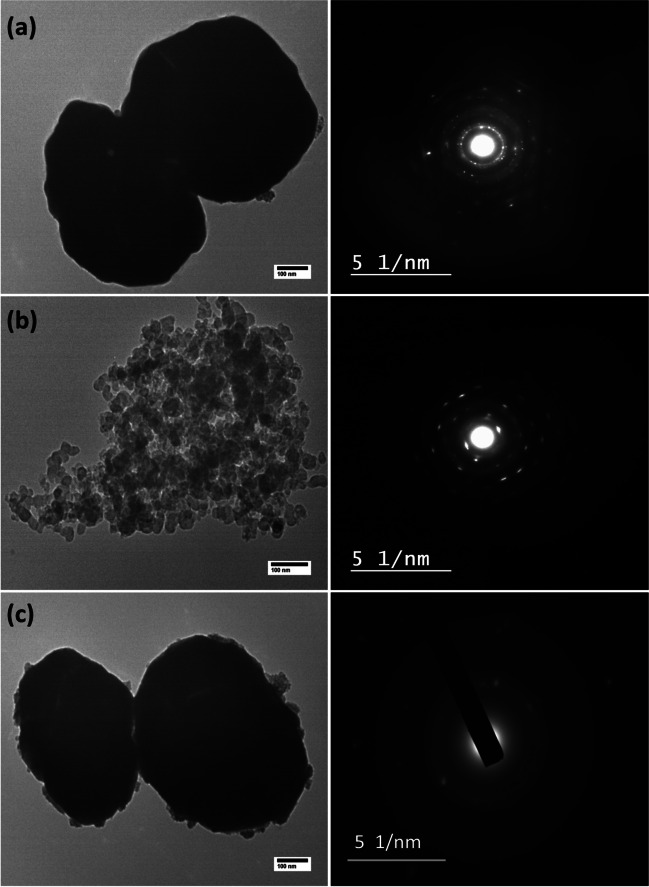
TEM images and SAED patterns of **a** Cu, **b** CuO, and **c** Cu/CuO nanoparticles

TEM images in Fig. 5(a) revealed the formation of agglomerated non-uniform Cu NPs with different particle sizes varying from 343 to 460 nm. The corresponding Selected Area Electron Diffraction (SAED) pattern recorded was a ring-like pattern confirming the highly crystalline nature of the synthesized Cu NPs (Nagar and Devra, 2018). The images in Fig. 5(b) show spherical CuO NPs with size distribution range of 20–31 nm which are consistent with XRD results and also with previous reports (Badri et al., 2021; Shah et al., 2022). The high surface energy of the synthesized CuO NPs causes aggregation. As shown in Fig. 5(c), Cu/CuO NPs were largely uniform and spherical in shape; this result agrees with the shape and uniformity of previously synthesized Cu/CuO nanoparticles (Khatami et al., 2017; Mohamed, 2020).

**Fig. 6:**
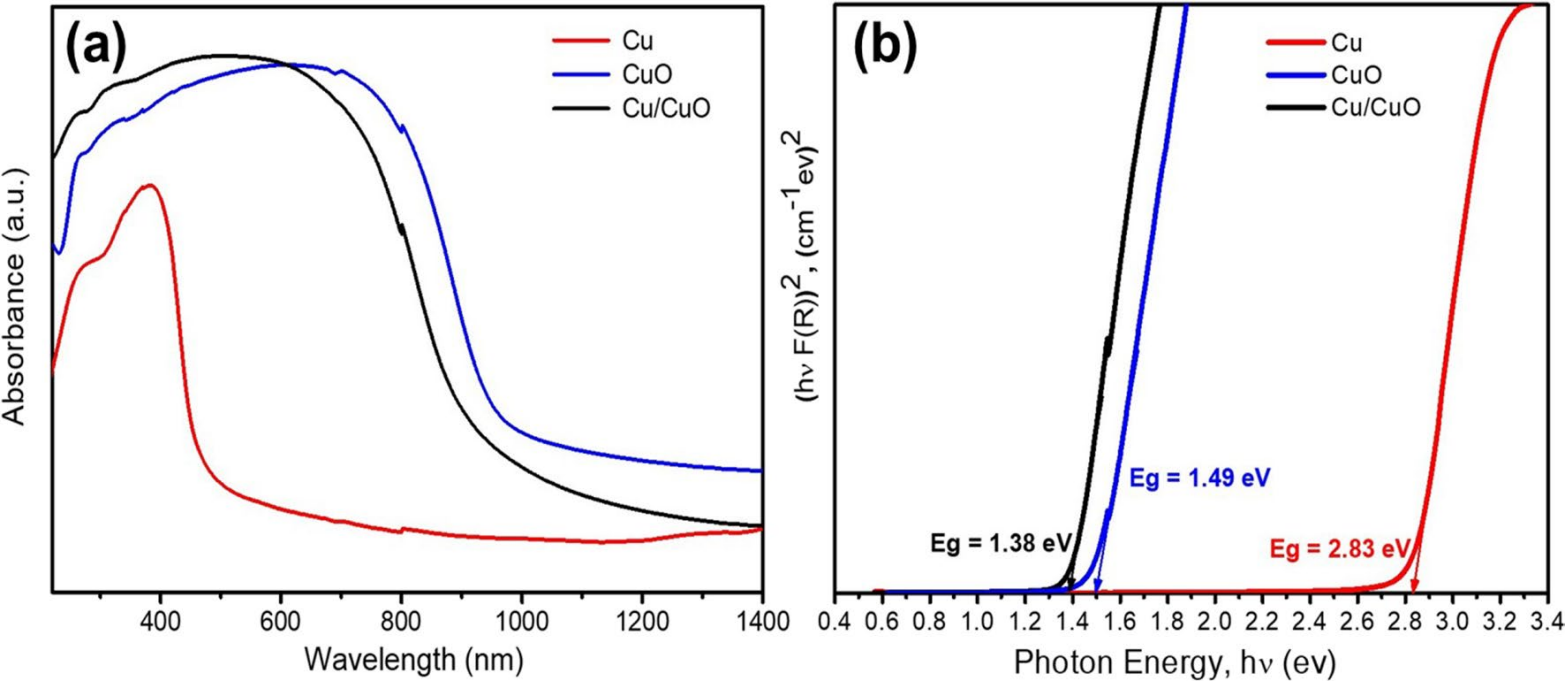
**a** UV-Visible spectrum and **b** optical band gap energy of Cu, CuO, and Cu/CuO nanoparticles

*Figure* *6**depicts the absorption peaks recorded for Cu, CuO, and Cu/CuO NPs* using a UV-Visible double-beam spectrophotometer*.* The absorption peak edges of *Cu, CuO, and Cu/CuO blend nanoparticles* are observed at 403, 680, and 850 nm, respectively, which correspond to the distinguishing peak of copper ions (Cu^2+^ and Cu^+^ ions). However, the ***absorption peak*** below 850 nm confirmed the highly crystalline and monophase of CuO nanoparticles. *These values are in good agreement with the reported results for copper and copper oxide nanoparticles* *(Abbasi-Kesbi et al.,* 2018*;* *Sahai et al.,* 2016*;* *Sudha et al.,* 2021*). The peak at 546 nm is ascribed to existence of Cu/CuO NPs (Bhattacharjee and Ahmaruzzaman,* 2015*;* *Dagher et al.,* 2014*). These findings are compatible with those found in literature (Sahai et al.,* 2016*;* *Swarnkar et al.,* 2009*;* *Vidyasagar et al.,* 2011*).* The data was used to calculate the samples’ indirect band gap values using Tauc plots, as shown in Fig. 6(b). The band gap values obtained from the linear intercept on the *x*-axis are ~2.38, 1.49, and 1.38 eV for Cu, CuO, and Cu/CuO blend, respectively, and the Cu/CuO blend is suitable for visible light absorbance. The value obtained is higher than the reported bulk values (Fuku et al., 2020; Nayak et al., 2020).

In the case of an infinitely powder samples, where thickness and sample holder have no influence on the reflectance (*R*) value, the Kubelka-Munk equation at any wavelength becomes (Yable et al., 2020)

5 $$F\left(R\infty \right)=\frac{\left(100-R\right)}{(2R)}$$

*F* (*R***∞**) is the Kubelka-Munk function. The band gap *E*_g_ and absorption coefficient *α* of a direct band gap semiconductor are interrelated through Eq. (6) (Abdelbasir et al., 2018a; Makuła et al., 2018):

6 $$\alpha h\nu =A{\left( h\nu -{E}_g\right)}^{1/n}$$

where *α* is the material’s linear absorption coefficient, *hν* is the photon energy, and *A* is a proportionality constant. When the material scatters evenly, the Kubelka-Munk absorption coefficient (*K*) equals 2*α* (*K* = 2*α*). In this case, bearing in mind the *K*-*M* scattering coefficient *S* as constant with respect to *λ*, and using the remission function in Eq. (5), we get the following equation (Rayan and Ismail, 2018):

7 $${\left( h\nu F\left(R\infty \right)\right)}^n=B\left( h\nu -{E}_g\right)$$

Thus, by plotting the [*F* (*R***∞**)*hν*]^2^ versus *hν*, the band gap *E*_g_ of a nanoparticles’ sample can be easily obtained. Figure 6(b) depicts the band gap energy of Cu, CuO, and Cu/CuO ***NPs*** after Kubelka-Munk treatment.

### Antibacterial activity testing

The antibacterial action of Cu NPs, CuO NPs, and (1:1) blend of Cu/CuO NPs against different strains of pathogenic bacteria are shown in Table 2. The most potent antibacterial action was exhibited by CuO NPs against *E*. *coli* with 21.2-mm zone of inhibition, followed by Cu NPs with inhibition zone 16.8 mm against *B. cereus*. While the lowest zone of inhibition 8.3 and 9.0 mm was detected against *P. aeruginosa* by CuO NPs and Cu NPs, respectively. On other hand, the mixture of Cu NPs and CuO NPs (1:1) had high antibacterial action against all tested pathogenic bacteria except *E. coli* with inhibition zone ranging between 10.2 and 16.7 mm. The negative control (DMSO) had no effect, whereas positive control, antibiotics ceftriaxone at concentration (1 mg mL^−1^), exhibited inhibition of 10.8 mm in *B*. *cereus*, 15.8 mm in *Staphylococcus aureus*, 34.8 mm in *E. coli*, 20.3 mm in *S*. *typhi*, and 16.3 mm in *P. aeruginosa*. Cu NPs are toxic to *E*. *coli* cells in a variety of ways including generation of reactive oxygen species, lipid peroxidation, protein oxidation, and DNA degradation (Chatterjee et al., 2014). CuO NPs have large surface area and are therefore extremely reactive (Nabila and Kannabiran, 2018). Having a high surface-to-volume ratio, copper nanoparticles interact directly with bacteria’s cell membranes, causing their deaths (Usman et al., 2013). Furthermore, Cu nanoparticles inhibit bacterial cell growth, which results in bactericidal effects (Nabila and Kannabiran, 2018).

**Table 2:** Antibacterial activity of Cu, CuO, and Cu/CuO NPs against pathogenic bacteria

Bacteria	Negative control	Positive control	Cu	CuO	(Cu/CuO) blend (1:1)
*B. cereus*	0	10.8 ± 1.04	16.8 ± 1.53	10.8 ± 1.25	16.7 ± 0.28
*Staphylococcus aureus*	0	15.8 ± 1.44	12.7 ± 1.04	11.2 ± 0.76	16.3 ± 1.04
*E. coli*	0	34.8 ± 1.25	10.3 ± 0.76	21.2 ± 2.56	11.3 ± 1.04
*S. typhi*	0	20.3 ± 1.04	9.3 ± 0.76	9.3 ± 1.25	10.2 ± 0.76
*P. aeruginosa*	0	16.3 ± 2.25	9.0 ± 1.00	8.3 ± 0.76	15.7 ± 1.46

*n* = 3

*SE*: standard error, *negative control*: DMSO, *positive control*: ceftriaxone

Table 3 shows the antifungal activity of Cu NPs, CuO NPs, and the (1:1) blend of Cu/CuO NPs against different strains of mycotoxigenic fungi. The strongest antifungal action was demonstrated against *A. niger* by Cu NPs with inhibition zone 15.0 mm, followed by the mixture of Cu/CuO (1:1) blend NPs against *P. verrucosum* with 13.8-mm zone of inhibition. Whereas the lowest inhibition zone value 8.5 mm was recorded by CuO NPs against *P. verrucosum*. The same trend in bacteria was repeated in fungi; the blend of Cu/CuO NPs (1:1) showed high antifungal activity against all tested mycotoxigenic fungi except *A. niger* with inhibition zone values ranging from 12.0 to 13.8 mm.

**Table 3:** Antifungal activity of Cu, CuO, and Cu/CuO NPs against mycotoxigenic fungi

Fungi	Negative control	Positive control	Cu	CuO	(Cu/CuO) blend (1:1)
*A. flavus*	0	17.8 ± 2.56	11.8 ± 1.89	10.5 ± 1.00	12.0 ± 0.50
*A. niger*	0	20.7 ± 1.61	15.0 ± 1.32	12.2 ± 0.76	12.3 ± 1.04
*A. ochraceus*	0	19.7 ± 1.04	9.8 ± 1.04	9.0 ± 1.32	12.7 ± 1.15
*F. proliferatum*	0	9.2 ± 0.28	8.8 ± 0.68	10.3 ± 0.76	13.0 ± 1.32
*P. verrucosum*	0	19.8 ± 1.75	10.0 ± 1.32	8.5 ± 0.50	13.8 ± 0.76

*n* = 3

*SE*: standard error, *negative control*: DMSO, *positive control*: miconazol

Numerous studies have found Cu NPs to be antimicrobial against a variety of fungi (El-Shewy, 2019; Eslami Chalandar et al., 2017; Usman et al., 2013). A CFU method evaluation of copper oxide nanoparticles showed a decrease in the growth of the *C. albicans* pathogen by 77.06. Using the disk diffusion method, the inhibition zone for *C. albicans* pathogen was 15.33, in agreement with the CFU method and further confirming the effective antifungal effect of CuO nanoparticles.

**Fig. 7:**
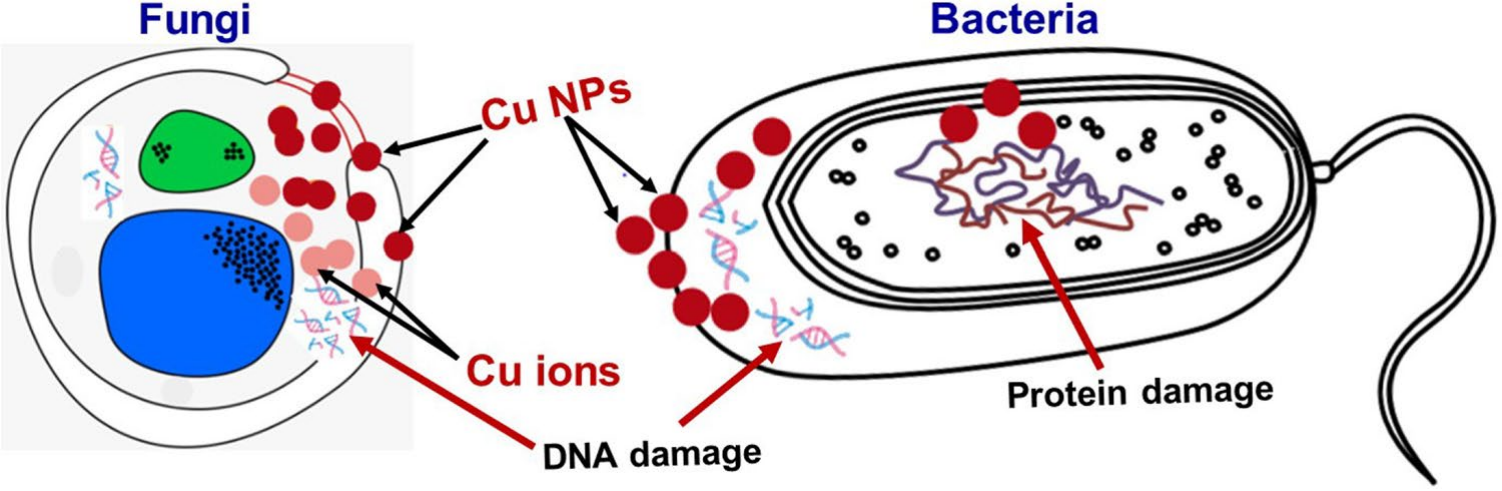
The main mechanism of bacteria and fungi death caused by Cu and CuO NPs

In general, nanoparticles inhibit microorganisms mainly by releasing the NPs and copper ions (Wang et al., 2014). The nanoparticles’ antimicrobial process produces reduced oxygen species (ROS; Dutta et al., 2015), destroys cell walls and membranes (Omid Akhavan and Ghaderi, 2010), and reacts with proteins and DNA (Kumar et al., 2011). In this process, copper-containing NPs can damage different microbial cell components through a variety of mechanisms. CuO NPs enhance bacterial activity by providing better contact with microorganisms. Cu ions released later, on the other hand, may also damage the DNA by binding with it (Salah et al., 2021), leading to total helical structure damage by cross-linking within and between the nucleic acid strands, as some researchers have suggested (Kumar et al., 2011; Malandrakis et al., 2019). The main mechanism of bactericidal activity is the generation of ROS, both dependent and independent of Fenton chemistry, and results in membrane damage (Wang et al., 2014). Cu NP influx is considered the primary mechanism in fungi resulting from ion uptake and physical deterioration of membranes. The activity of Cu NPs appears to depend more on their size rather than their concentration: the smaller the nanoparticles, the greater their efficiency. CuO NPs play an important role in cytoplasmic damage in fungi, which leads to the apoptotic nature of fungal strains. The main causes of bacteria and fungi death are presented in Fig.7. The performance comparison of Cu-based nanoparticles’ antibacterial activity with previous reports is shown in Table S1 in the supplemental file.

The results clearly show that eco-friendly synthesized CuO NPs can outperform commercially available standards as fungicidal agents. Figure S3 in the supplementary file exhibits the zone of inhibition of Cu, CuO, and Cu/CuO (1:1) blend nanoparticles, using DMSO and ceftriaxone as negative and positive controls, against various five bacteria and fungi strains. Table S1 shows a comparison of various copper-based photocatalysts in degradation of Rhod-B dye.

### Photocatalytic activity evaluation

**Fig. 8:**
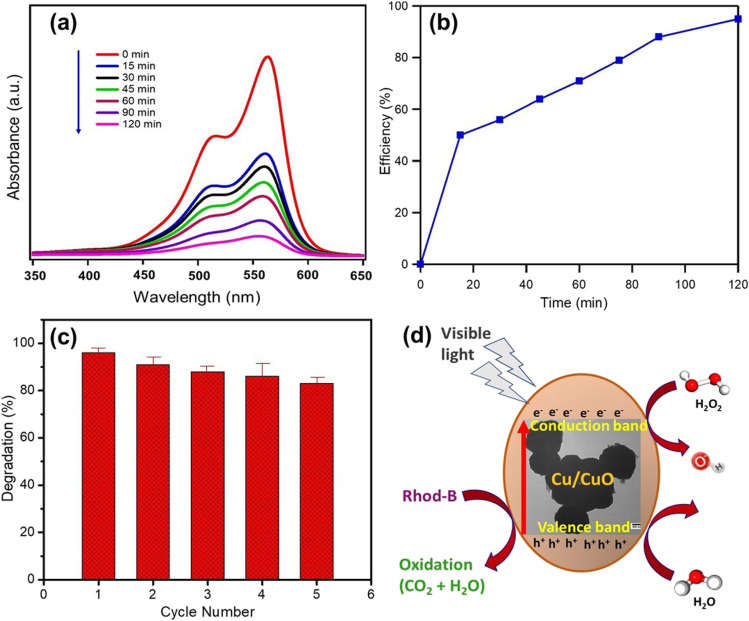
**a** Time-dependent absorbance spectra, **b** degradation efficiency, **c** cycling runs for the catalytic degradation of Rhod-B solution in the presence of the Cu/CuO photocatalyst, and **d** schematic illustration of Rhod-B dye degradation under visible light illumination

Absorbance spectra of Rhod-B after light irradiation to different time in presence of Cu/CuO photocatalyst are displayed in Fig. 8(a). With illumination time, all dyes’ absorption spectra gradually reduce and nearly disappear after 120 min, as seen in the absorbance spectra. The decrease in the concentration with irradiation of the dye can be clearly seen and the degradation was found to be about 97%, over a period of 120 min (Fig. 8(b)).

The chemical structure of the Rhod-B has a significant impact on the photodecolorization yield from a compositional standpoint. The azo group N = N can be photodecolorized. Besides having a high adsorption yield, Rhod-B with sulfonic groups displays increased dye reactivity when N = N is present. This is in line with previous research by Aljamali and colleagues (Aljamali, 2015). When Cu/CuO was added to the Rhod-B solution, the degradation started. A curve representing the absorption spectra of Rhod-B without Cu/CuO particles is shown at *t* = 0 min. When Cu/CuO particles are added, the intensity of the adsorption band at 553 nm decreases. It indicates that the absorption of Rhod-B dye by Cu/CuO particle and the potential breakdown of the azo bond causes the solution decolorization. The BET specific surface area of Cu/CuO particles was 5.0 m^2^ g^−1^ as measured by the BJH plot (see Table S2 in the supplemental file). The Cu/CuO NP blend exhibited a type III isotherm with a type H3 hysteresis loop according to the IUPAC classification, which indicates that the internal porosity of the formed powders consisted of slit-like and panel-like pores (Aftab et al., 2019).

The photocatalyst’s cyclic stability and reusability are always critical. As shown in Fig. 8(c), the stability of the Cu/CuO photocatalyst was determined over five cycles. After each cycle, the photocatalyst was separated from the dye solution by centrifugation, washed, redispersed, and reused in the following cycle. The duration of light irradiation for each cycle was 120 min, while all other experimental parameters remained constant. After five cycles, the Cu/CuO photocatalyst had 83.88% stability. As a result, the waste-derived material is regarded as a highly efficient and stable photocatalyst for Rhod-B dye breakdown.

### Mechanism of the dye degradation

It can be proposed that photodegradation occurs when visible light is irradiated on Cu/CuO blends, resulting in the production of active radicals at large band gaps for both CuO (1.49 eV) and Cu (2.83 eV). The excited electrons at the conduction band (CB) and holes at the valence band (VB) cause the generation of hydroxyl (^•^OH) and superoxide (^•^O_2_^**−**^) radicals. These active radicals are responsible for the breakdown of harmful organic dye molecules (Ranjith et al., 2018). Since Cu and CuO have different band gap values, the photoexcited electrons travel from the high-energy band edge to the lower energy band edge of individual material (Li et al., 2017; Mosleh et al., 2018).

CuO is a well-known semiconductor having a low band gap that absorbs visible light to generate electron-hole pairs. The presence of Cu NPs promotes photocatalytic efficiency, which can be explained by surface plasmon resonance (SPR) Cu NP increases visible light absorbance and helps improve the catalyst efficiency (Cheng et al., 2016). Furthermore, Cu has a low work function and is a good conductor, resulting in simple electron transport and a decrease in recombination. CuO’s CB potential was lower than the standard redox potential of O_2_/O_2_^−^, but higher than the standard redox potential of OH/OH^−^. As a result, starting the Cu/CuO NP photocatalytic process with visible light excites the free electrons and hole pairs at the catalyst surface. Superoxide ions are produced when dissolved oxygen reacts with conduction band electrons. The photocatalysis is carried out by the ^.^OH which is prohibited to be formed directly from H_2_O. There is a possibility that the holes on the surface of CuO can directly oxidize the dye and cause it to degrade (Barzegar et al., 2019). Cu particles, on the other hand, are excellent electron traps (Li et al., 2010; Liu et al., 2013; Phutanon et al., 2018) which further facilitates the separation of photo-induced charge carriers and greatly enhances the catalyst stability. A schematic illustration of Rhod-B dye degradation under visible light illumination is shown in Fig. 8(d). Table 4 compares various copper-based photocatalysts reported for the degradation of Rhod-B dye.

**Table 4:** Comparison of various copper-based photocatalysts in degradation of Rhod-B dye

Photocatalyst	Degradation time	Irradiation light	Degradation efficiency (%)	Reference
CuO NPs	100 min	Visible light	98.8–99.6	Dodoo-Arhin et al. (2021)
CuO NPs	100 min	UV	98.31	Rafique et al. (2020)
CuO nanofiber	160 min	Visible light	96	Zeng et al. (2018)
CuO//ZnO	180 min	Sunlight	98	Truong et al. (2021)
Flower-like CuO	240 min	UV	NR	Phutanon et al. (2018)
***ZnO/CuO/Ag***_***2***_***O***	105 min	Solar light	97.38	Meena et al. (2021)
Cu_2_O	200 min	UV	97	Kangralkar et al. (2021)
Cu_2_O/rGO	120 min	Visible light	95	Huang et al. (2017)
Cu/CuO	120	Visible light	96.5	***This work***

*NR*: not reported by the author

## Conclusion

*From e-waste as a starting material, Cu and CuO NPs were produced using eco-friendly methods.* The use of ammoniacal ammonium salt leaching to preferentially retrieve copper from WPCBs with a high yield is proposed. *The NPs were confirmed by XRD, FTIR, and UV-Visible analyses. TEM images exposed that the average particle size for Cu and CuO NPs was 460 nm and 50 nm, respectively. The as-produced NPs were tested for their potent antibacterial activity against five different bacterial and fungul pathogens. A 1:1 blend of Cu/CuO nanoparticles exhibited good bactericidal activity when compared to Cu and CuO alone. Moreover, the naoparticle blend was used as a photocatalyst for the degradation of rhodamine B (Rhod-B) dye under visible light illumination. The blend showed excellent decomposition of Rhod-B at 120 min with an efficiency of 96.5%, which is due to the lower energy band gap of 1.3 eV compared to 1.49 eV and 2.38 eV for Cu and CuO, respectively. As a result, the WPCB-derived NPs can be used as an effective antibacterial agent and photocatalyst in a variety of textile and food industries.* Lastly, based on the previously mentioned results, the developed strategy appears to have the potential to be a reliable source of both Cu and CuO nanoparticles, as well as many other nanoparticles with various sizes and shapes, since WPCBs contain several metals aside from Cu, such as Sn, Ag, and Au. Further, the recovered metals can be applied for other applications like photochemical catalysis, optics, gas sensors, solar energy conversion, and electronic industry, where mineral resources are in short supply.

## Supplementary information

ESM 1: **Fig. S1.** a schematic drawing of the used leaching system. **Fig. S2.** System used for nanoparticles’ synthesis. **Fig. S3.** Bactericidal activity of Cu, CuO and Cu/CuO NPs against (a) five pathogenic bacteria (using DMSO, ceftriaxone as negative and positive controls), and (b) five mycotoxigenic fungi (using DMSO and miconazol as negative and positive controls). **Table S1.** The performance comparison of Cu-based nanoparticles antibacterial activity with previous reports. **Table S2.** BET surface area (S_BET_), pore size (d), and pore volume (Vg) of Cu/CuO NPs blend.

## Data Availability

The authors confirm that all data supporting the study’s findings are included in the article.

## Declarations

Ethics approval and consent to participate
