# Supplementary material for: Synthesis of Cu and CuO nanoparticles from e-waste and evaluation of their antibacterial and photocatalytic properties
Source: Environ Sci Pollut Res Int. 2023 Jul 17;30(38):89690–704. doi: 10.1007/s11356-023-28437-5 (PMC10412494; doi:10.1007/s11356-023-28437-5)
Supplement: Supplementary file 1 — Fig. S1. a schematic drawing of the used leaching system. Fig. S2. System used for nanoparticles’ synthesis. Fig. S3. Bactericidal activity of Cu, CuO and Cu/CuO NPs against (a) five pathogenic bacteria (using DMSO, ceftriaxone as negative and positive controls), and (b) five mycotoxigenic fungi (using DMSO and miconazol as negative and positive controls). Table S1. The performance comparison of Cu-based nanoparticles antibacterial activity with previous reports. Table S2. BET surface area (SBET), pore size (d), and pore volume (Vg) of Cu/CuO NPs blend. [file 11356_2023_28437_MOESM1_ESM.docx]

**Synthesis of Cu and CuO Nanoparticles from E-waste and Evaluation of Their Antibacterial and Photocatalytic Properties**

Sabah M. Abdelbasir^1,*^, Diaa A. Rayan^1,3^, Mahmoud M. Ismail^2^

*^1^Central Metallurgical R& D Institute (CMRDI), P.O. Box 87 Helwan 11421, Cairo, Egypt*

*^2^Physics Dept., Faculty of Science, Al-Azhar Unversity, Nasr City, Cairo 11884, Egypt*

*^3^Physics Dept, Deraya University, New Minya, Minya, Egypt*

*Corresponding author: [sfoda20@hotmail.com](mailto:sfoda20@hotmail.com), [sfoda@cmrdi.sci](mailto:sfoda@cmrdi.sci). eg

*Tel.: +202 27142011; +201151195565; fax: +202 27142451.*

ORCID/ 0000-0003-2698-2041


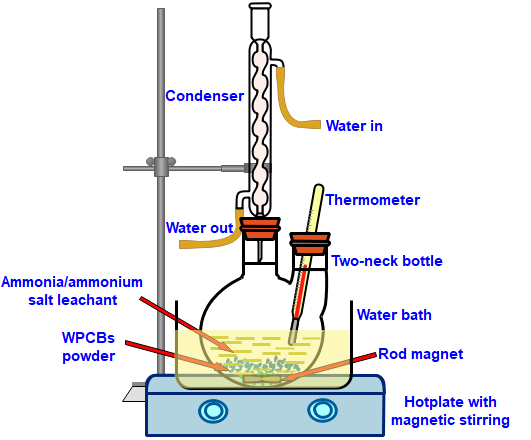


**Fig. S1.** a schematic drawing of the used leaching system.


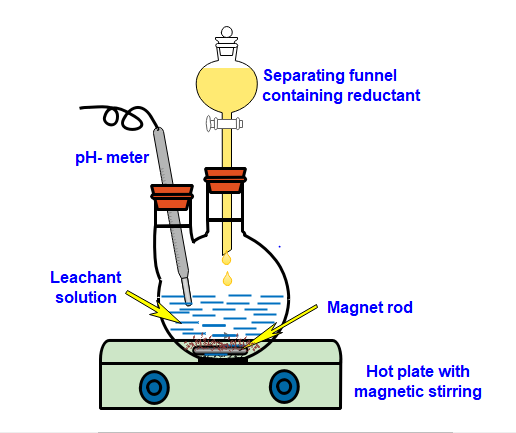


**Fig. S2.** System used for nanoparticles’ synthesis.

***Materials Characterization***

X-ray diffractometer (XRD) “Bruker AXS- D8, Germany” in the range 2θ from 20 to 70^o^ using CuKα as a radiation source (λ = 1.54 Å). Fourier Transform Infrared Spectroscopy (FT-IR) was accomplished by means of JASCO-3600 spectrometer over the wavenumber spans from 4000-400 cm^-1^. Transmission electron microscope (TEM) “JEOL-JEM1230” was used for microscopic structural and morphology investigation of the synthesized nanoparticles. The absorbance spectra of the prepared particles were distinguished by a UV/Vis spectrophotometer Varian Cary 100 Scan system at 190–2200 nm with BaSO_4_ as a reference.


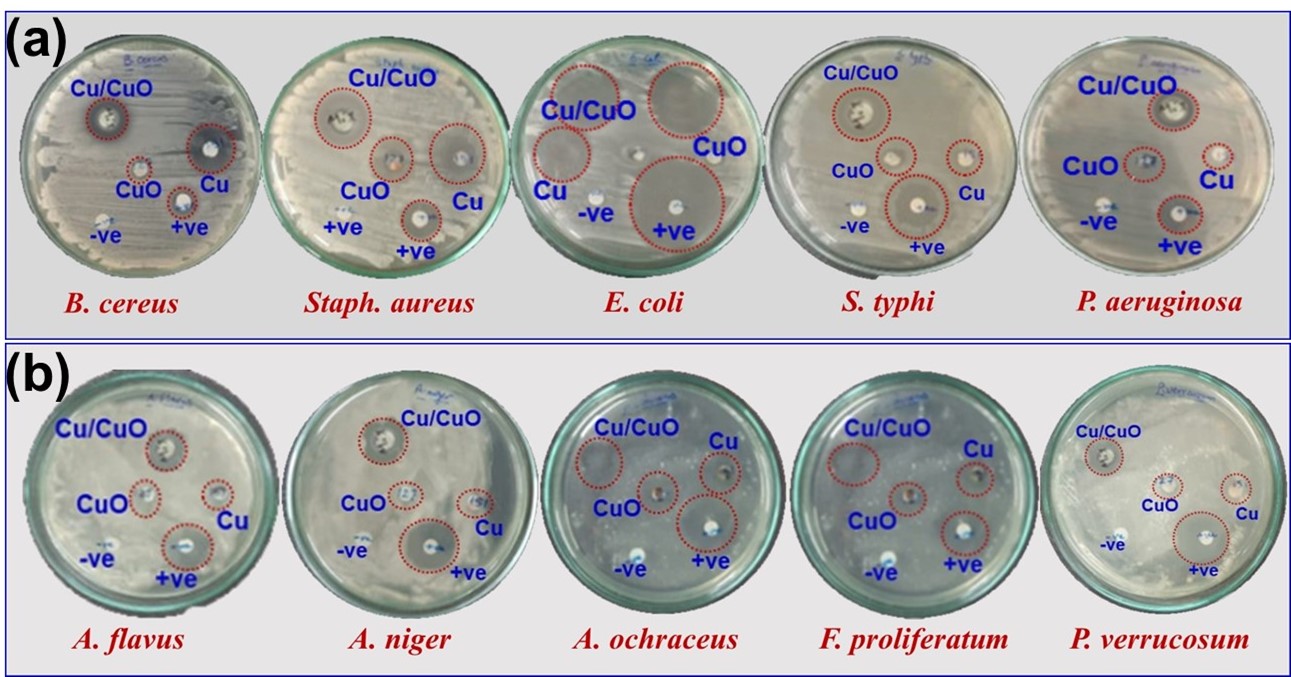


**Fig. S3.** Bactericidal activity of Cu, CuO and Cu/CuO NPs against (a) five pathogenic bacteria (using DMSO, ceftriaxone as negative and positive controls), and (b) five mycotoxigenic fungi (using DMSO and miconazol as negative and positive controls).

**Table S1.** The performance comparison of Cu-based nanoparticles antibacterial activity with previous reports

| **Nanoparticles** | **Precursor** | **Bacterial strain** | **Control** | **Inhibition zone (mm)** | **Reference** |
| --- | --- | --- | --- | --- | --- |
| Cu/Cu_2_O NPs | Copper chloride (CuCl_2_. 2H_2_O) and *S. lavandulifolia* flower | P. aeruginosa | Streptomycin | 12 | (Khatami et al., 2017) |
| CuO NPs | Copper (II) sulfate pentahydrate (CuSO_4_ .5H_2_O) and *Solanum trilobatum* Extract | *Enterococcus aerogenes, Staphylococcus aureus, Escherichia Coli, Pseudomonas aeruginosa,*and *Proteus vulgaris* | Amoxicillin | 2.0-4.0 | (Bharathy et al., 2021) |
| CuO NPs | Copper (II) acetate and sodium hydroxide | *Escherichia coli*, *Pseudomonas aeruginosa*, *Klebsiella pneumonia*, *Enterococcus faecalis*, *Shigella flexneri*, *Salmonella typhimurium*, *Proteus vulgaris,* and *Staphylococcus aureus* | **Streptomycin** | 5-20 | (Ahamed et al., 2014) |
| CuO NRs | Copper (II) sulfate pentahydrate (CuSO_4_.5H_2_O) and Momordica charantia (MC) fruit | (Staphylococcus aureus, Streptococcus mutans, Streptococcus pyogenes, Streptococcus viridans, Staphylococcus epidermidis, Corynebacterium xerosis, and Bacillus cereus) and (Escherichia coli, Klebsiella pneumonia, Pseudomonas aeruginosa, and Proteus vulgaris) | Streptomycin  and Norfloxacin | 23-31 | (Qamar et al., 2020) |
| Cu_2_O NPs | CuSO_4_.5H_2_O and NaBH_4_ | *Pseudomonas aeruginosa* and *Bacillus subtilis* | ^*^NR | 14.6-28.6 | (Bezza et al., 2020) |
| CuO NPs | CuSO_4_.5H_2_O and Cedrus deodara | S. aureus and E. coli | ^*^NR | 29 | (Ramzan et al., 2021) |
| CuO NPs | CuNO_3_·2H_2_O and apple extract | S. aureus and E. coli | ^*^NR | 26.0 and 29 | (Rajamohan et al., 2023) |
| Cu NPs  (Cu-embedded agar film) | CuSO_4_.5H_2_O and modified dialdehyde starch (DAS) | E. coli | bare agar film | No ^**^CFUs were found in the incubation plate | (Tang et al., 2018) |
| Cu NPs | Copper acetate hydrate (CuAc_2_.2H_2_O), Tween 80 (polyoxyethylene-(80)-sorbitan monooleate) and ethylene glycol | Micrococcus luteus, Staphylococcus aureus , Escherichia coli, Klebsiella pneumoniae, and Pseudomonas aeruginosa | penicillin G | 5-26 | (Ahamed et al., 2014) |
|  |  |  |  |  |  |
| Cu/CuO | WPCBs and L. ascorbic acid | *Bacillus cereu*, *Staphylococcus aureus*, *Salmonella typhi*, *Escherichia coli, and Pseudomonas aeruginosa* | DMSO and ceftriaxone | 8.3-21.2 | ***This work*** |

^*^NR: not reported by the author, ^**^CFU: colony-forming units

**Table S2.** BET surface area (S_BET_), pore size (d), and pore volume (Vg) of Cu/CuO NPs blend.

| **S_BET_ (m^2^ g^−1^ )** | **Average pore size, d (nm)** | **Pore volume, Vg (cm^3^ g^−1^)** |
| --- | --- | --- |
| 5.0 | 222.98 | 0.063314 |

**Supporting references**

Ahamed M, Alhadlaq HA, Khan MAM, Karuppiah P, Al-Dhabi NA. Synthesis, characterization, and antimicrobial activity of copper oxide nanoparticles. J Nanomater 2014;2014. https://doi.org/10.1155/2014/637858.

Bezza FA, Tichapondwa SM, Chirwa EMN. Fabrication of monodispersed copper oxide nanoparticles with potential application as antimicrobial agents. Scientific Reports 2020 10:1 2020;10:1–18. https://doi.org/10.1038/s41598-020-73497-z.

Bharathy MS, Jeyaleela GD, Vimala JR, Agila A, Hemadevi M. Potential Use of Copper Oxide Nanoparticles from Solanum Trilobatum Against Pathogenic Bacteria. Oriental Journal Of Chemistry 2021;37:991–6. https://doi.org/10.13005/OJC/370429.

Khatami M, Heli H, Jahani PM, Azizi H, Nobre MAL. Copper/copper oxide nanoparticles synthesis using Stachys lavandulifolia and its antibacterial activity. IET Nanobiotechnol 2017;11:709–13. https://doi.org/10.1049/IET-NBT.2016.0189.

Qamar H, Rehman S, Chauhan DK, Tiwari AK, Upmanyu V. <p>Green Synthesis, Characterization and Antimicrobial Activity of Copper Oxide Nanomaterial Derived from <em>Momordica charantia</em></p>. Int J Nanomedicine 2020;15:2541–53. https://doi.org/10.2147/IJN.S240232.

Rajamohan R, Raorane CJ, Kim SC, Lee YR. One Pot Synthesis of Copper Oxide Nanoparticles for Efficient Antibacterial Activity. Materials 2023;16:217. https://doi.org/10.3390/MA16010217/S1.

Ramzan M, Obodo RM, Mukhtar S, Ilyas SZ, Aziz F, Thovhogi N. Green synthesis of copper oxide nanoparticles using Cedrus deodara aqueous extract for antibacterial activity. Mater Today Proc 2021;36:576–81. https://doi.org/10.1016/J.MATPR.2020.05.472.

Tang L, Zhu L, Tang F, Yao C, Wang J, Li L. Mild Synthesis of Copper Nanoparticles with Enhanced Oxidative Stability and Their Application in Antibacterial Films. Langmuir 2018;34:14570–6. https://doi.org/10.1021/ACS.LANGMUIR.8B02470/SUPPL_FILE/LA8B02470_SI_001.PDF.
